# *Erythrina suberosa*: Ethnopharmacology, Phytochemistry and Biological Activities

**DOI:** 10.3390/medicines6040105

**Published:** 2019-10-18

**Authors:** Felicia Patti, Yasaman Taheri, Javad Sharifi-Rad, Miquel Martorell, William C. Cho, Raffaele Pezzani

**Affiliations:** 1 OU Endocrinology, Dept. Medicine (DIMED), University of Padova, via Ospedale 105, 35128 Padova, Italy; feliciapatti@hotmail.com; 2 Phytochemistry Research Center, Shahid Beheshti University of Medical Sciences, Tehran 11369, Iran; 3 Department of Pharmacology and Toxicology, School of Pharmacy, Shahid Beheshti University of Medical Sciences, Tehran 11369, Iran; 4 Zabol Medicinal Plants Research Center, Zabol University of Medical Sciences, Zabol 61615-585, Iran; 5 Department of Nutrition and Dietetics, Faculty of Pharmacy, University of Concepcion, Concepcion 4070386, Chile; martorellpons@gmail.com; 6 Universidad de Concepción, Unidad de Desarrollo Tecnológico, UDT, Concepción 4070386, Chile; 7 Department of Clinical Oncology, Queen Elizabeth Hospital, 30 Gascoigne Road, Hong Kong; 8 AIROB, Associazione Italiana per la Ricerca Oncologica di Base, 35128 Padova, Italy

**Keywords:** *Erythrina suberosa*, *Erythrina* genus, phytopharmacology, anticancer

## Abstract

Plants are a great and irreplaceable source of medicines, fuel, food, energy and even cosmetics. Since prehistory, humans have learned to use plants for survival, growth and proliferation and still today it relies on natural and cultivated vegetables for food and the source of novel compounds with pharmacological activity. Not only herbs and flowers, but also trees are used. Indeed, *Erythrina suberosa* Roxb. is a deciduous tree of the family Fabaceae, common in Southeast Asia. In India, *E. suberosa* is called the “corky coral tree” or simply the “Indian coral tree”, given its peculiar red-orange flowers that can flower throughout the year and its corky irregular bark covered by prickles. It is a plant commonly used as an ornamental tree, but it also holds ethnopharmacological and socioeconomic uses. This article explored phytobiological features of *E. suberosa*, analysing its taxonomy, examining its traditional and common uses and investigating its bioactive components and pharmacological properties.

## 1. Introduction

*Erythrina suberosa* Roxb. is a deciduous tree of medium size (about 10 m) belongs to the family Fabaceae. Native of Punjab region and called “Pangra” [1], it is widely used in Pakistan and India as an ornamental tree. *E. suberosa* belongs to the genus *Erythryna* which comprises more than 100 species: according to Da Silva, et al. [2], the *Erythryna* genus includes 120 species distributed in Tropical and Subtropical regions. The appellation “coral tree or flame tree” is commonly given to the *Erythryna* genus because of its red flowers and because the branches can resemble the shape of sea coral [3]. Furthermore, the origin of the name comes from the Greek “erythros”, which truly means “red”. According to the Annotated Checklist of the Flowering Plants of Nepal [4] and ILDIS [5], *E. suberosa* can be used as a synonym for *Erythrina glabrescens* (Prain) R. Parker. This review article focuses on the species *E. suberosa*, analysing its taxonomy, traditional and common uses, bioactive components and pharmacological properties. Many other works have analyzed the properties and effects of the *Erythrina* genus and we suggest that the reader examine subsequent pivotal reviews [6,7].

## 2. Taxonomy and Morphologic Description

*E. suberosa* belongs to the kingdom of Plantae (Plants), division of Magnoliophyta (flowering plants), class of Magnoliopsida (Dicotyledons), order of Fabales, family of Fabaceae (Legume family), subfamily of *Papilionoideae,* genus of *Erythrina* (L.), species *suberosa* (Roxb.) [8]. In addition to the accepted name, *E. suberosa* presents many nonpreferred scientific names, such as *Erythrina alba* Wight& Arn., Micropteryx sublobata (Roxb.) Walp., *Erythrina glabrescens* (Prain) R.Parker, *Erythrina maxima* Wight & Arn., *Erythrina stricta* var. suberosa (Roxb.), *Erythrina sublobata* Roxb., and *Micropteryx suberosa* (Roxb.) Walp. *E. suberosa* is commonly referred to in Hindi as “Pangra”, in Malayalam as “Mullumurukku”, while in Tamil it is referred to as “Mullumurungu” [9].

**Figure 1:**
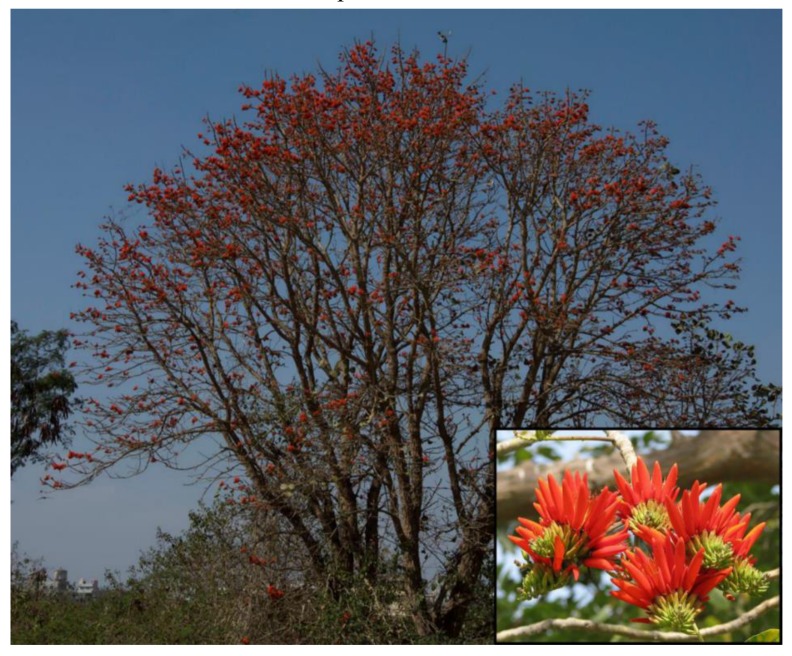
*Erythrina suberosa* Roxb. tree and flowers.

The *E. suberosa* tree is of medium size (can grow up to 10 m high) and has a grey, corky, deeply cracked bark (Figure 1). The branches have prickles, while branchlets are tomentose and vigorous [10]. The flowers are of bright scarlet, are 4 cm long, bisexual, and placed in axillary and terminal racemes which blossom February–April. The leaves are trifoliate and alternate with a leaflet size of 15–10 cm × 6–12 cm, a rhomboid-oval shape, and are glabrous above and woolly pubescent underneath. The fruits are pods that can easily reach 15 cm and fructify April–May. There are normally 2–5 seeds per fruit and these can be subdivided into two types: (a) light brown (b) dark reddish-brown seeds. This colour dimorphism of the seeds can be seen within a single pod, not only in an individual plant. They have a shape of an ellipsoid (reniform), and they are smooth and glittering.

*E. suberosa* is widely distributed in plain regions of India (common around Pune), Nepal, Bhutan, Burma, Thailand and Vietnam, sporadically on hills and on moist slopes [11,12]. Although the number of *Erythrina* species is large worldwide, in India there is a limited number of plants belonging to *Erythrina* genus, such as *E. suberosa*, *E. fusca* Lour., *E. resupinata* Roxb., *E. stricta* Roxb., *E. arborescens* Roxb., *E. subumbrans* (Hassk.) Merr., and *E. variegata* L. (syn. *E. indica* Lam.), which are all wild species. There are also cultivated species: *E. crista-galli* L., E. *corallodendrum* L., *E. mitis* Jacq., *E. poeppigiana* (Walp.) O.F. Cook., and *E. blakei* Parker [13]. It is notable that all of these reported species are also present in Andhra Pradesh, Pakistan and Nepal.

## 3. Traditional and Common Uses

Besides being an ornamental tree, *E. suberosa* is traditionally used in the Southeast of Asia as an ethnopharmacological source for numerous preparations.

In an ancient Indian traditional medicine called “Siddha”, *E. suberosa* has been used in many habitual formulations involving the consumption of all the plant, such as radix, leaves, bark, and flowers. In particular, radix can be used as emmenagogue, while leaves can be used as cathartics, anthelmintics, galactogenics and diuretics [14]. Paste made from the leaves can be applied to swellings and boils, given its antiseptic and anti-inflammatory effects [14]. Stem bark can have expectorant, bronchodilator, laxative, spasmolytic, anthelmintic, diuretic, and emmenagogue properties; indeed, bark decoction is used for dysentery, worm infestation and as eye lotion in opthalmia [15]. Moreover, the fresh juice is used topically for ulcers, wounds and sores [15]. Flowers are used to reduce nausea, for ear troubles and the aqueous extract can be mixed with *Hibiscus rosa-sinensis* L. as a soothing drink with a refreshing and relaxing effect during the summer season [16]. Conversely, the seeds are poisonous if ingested, but they are commonly used outside of the pharmacological field in different objects such as necklaces, rosaries and good-luck charms [17].

In traditional Tibetan medicine, *E. suberosa* flowers play a fundamental role in treating numerous fever-producing diseases, such as those afflicting the liver, lungs and heart [14].

*E. suberosa* is also employed in veterinary medicine. Indeed, bark ashes are mixed with coconut oil and applied to pustules, boils and wounds in cattle as an antiseptic; bark decoctions (stem without bark, crushed and extracted) are applied to swelling or injuries in animals [18]. It is reported that stem bark can be used as a fish poison: in addition, *E. suberosa* can have the ability to tint clothes (as a dark brown dye), it is used for the manufacture of cork and the wood from *E. suberosa* is employed in the production of light boxes and as fuel [18].

## 4. Bioactive Components and Phytoconstituents

**Figure 2:**
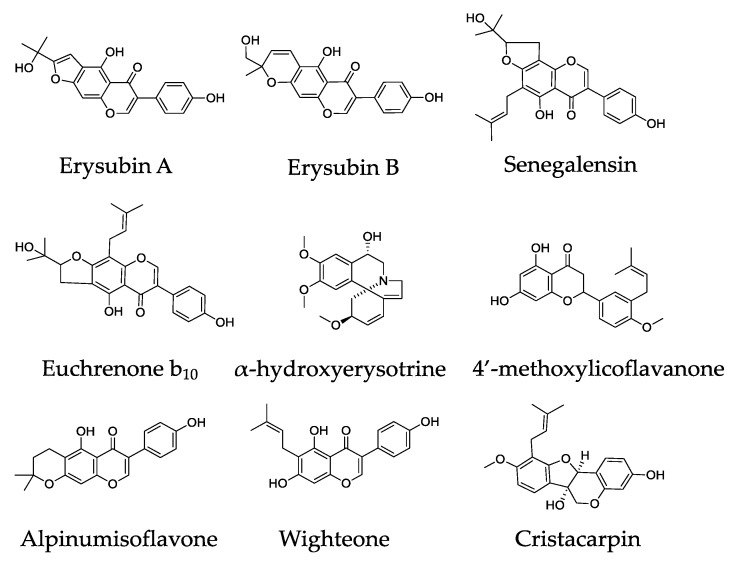
Chemical structures of selected compounds isolated from *Erythrina suberosa* Roxb.

Different works have analyzed the composition of *E. suberosa*. From the wood of *E. suberosa*, Tanaka [19] isolated two isoflavones—erysubins A and B—and more recently in 2001, the same group found two prenylated isoflavones, with hydroxyisopropyldihydrofuran moiety, senegalensin and its regioisomer euchrenone b_10_ in the wood of *E. suberosa* var. *glabrescence* [20] (Figure 2). In addition, from the bark of *E. suberosa*, a more recent work has isolated four different metabolites, i.e., α-hydroxyerysotrine, 4′-methoxylicoflavanone (MLF), alpinumisoflavone (AIF) and wighteone [21]. Furthermore, the roots of *E. suberosa* contain different alkaloids as well as prenylated flavonones and isoflavonoids, i.e., erysubin C (a pterocarpan with a formile group), erysubins D-E-F and cristacarpin [20,22]. Moreover, from the seeds of *E. suberosa* (which contain a high number of organic acids, alkaloids and steroids), Singh and Chawla [23] isolated different alkaloids such as erytraline, erysodine, erysotrine and hypaphorine. For the first time, erysotrine (usually found as a product of other eryso-alkaloids), an alkaloid present in many plants, was found to occur naturally in *E. suberosa* [22]. Also, it has been shown that the oil extracted from the seeds contains fatty acids such as sitosterol, stigmasterol, campesterol and cholesterol [22]. Again, in the seeds, Bhattacharyya, et al. [24] discovered the presence of D-galactose-binding lectins.

From the leaves of *E. suberosa*, similarly to the seeds, erysotrine was isolated and characterized [25], while from the flowers (crude extract), tannins, anthraquinones, sterols, terpenes, flavonoids, saponins and phenolic compounds were isolated [16].

## 5. Pharmacological Properties

To our knowledge, no work has examined the effects of *E. suberosa* or the genus *Erythryna* on humans, although many studies were conducted on preclinical models. Most of the works in the literature deal with the anticancer properties of *E. suberosa*, spanning from haematological to solid malignancies and from human derived cancer cells to mouse models [26]. Dhar, et al. [27] first evaluated the effects of a water–ethanol extract (1:1) of *E. suberosa* leaves (and many other Indian plants). They showed that *E. suberosa* could have anti-neoplastic and potential pharmacological activity in Sarcoma 180 (*Mus musculus* sarcoma), but no other indications were added [27]. Another work demonstrated that *E. suberosa* leaf extract had no anticancer effects on L1210 cells (*Mus musculus* skin lymphocytic leukemia) [21] probably underlining that different extraction methods could influence pharmacological properties.

Additionally, human cancer cell lines were investigated: HL60 (human promyelocytic leukemia) cells were tested with *E. suberosa* bark ethanolic extract [28]. Cell proliferation was inhibited in a dose- and time-dependent manner and the cell cycle was modulated by an increase in subG0/G1 cell phase. Moreover, an increase in cytosolic cytochrome C was shown with a concomitant intrinsic apoptosis pathway activation, with the stimulation of caspases 9 and 3, but not caspase 8. The authors suggested that *E. suberosa* extract could induce a mitochondria-mediated intrinsic apoptotic pathway and potentially could have a role in treating human leukemia.

Similarly, Kumar, Pathania, Saxena, Vishwakarma, Ali and Bhushan [3] isolated four metabolites from *E. suberosa* bark extract (MLF, AIF, wighteone, α-hydroxyerysotrine) and tested them on HL60 cancer cells. They showed that both MLF and AIF demonstrated the most potent cytotoxicity with IC_50_ of ~20 µM in HL60 cells and could induce apoptosis through inhibition of NF-kB factor (through the signal transducer of activation (STAT) pathway) [3]. Moreover, wighteone, an isoflavone derived from *E. suberosa* stem bark as mentioned above, demonstrated anticancer effects in MCF-7 HER2-positive cells (human mammary cancer cells) mediated by the heat shock protein 90 (HSP90), again increasing apoptosis and reducing cell proliferation [29]. Is it notable that wighteone can be found in different plants: *Cudrania tricuspidata* (Carrière) Bureau, *Ficus tikoua* Bur., *Maclura pomifera* (Raf.) C.K. Schneid. [30,31] and in different species of the genus *Erythrina*: *stricta, fusca, poeppigiana* and *variegata* [32,33,34,35].

Furthermore, a work on cristacarpin, a prenylated pterocarpan found in *E. suberosa* bark, confirmed that both PANC-1 (human pancreatic cancer cells) and MCF-7 cells were sensitive to this compound. Indeed, cell viability was decreased without toxic effects on non-cancer cell lines, such as HUVEC (human endothelial cells) and BPH-1 cells (human benign prostatic hyperplasia cells) [36]. In addition, the authors showed that cristacarpin, in an in vivo experiment with a 4T1 allograft mouse mammary carcinoma model, could prevent tumor growth by inducing premature senescence through both G1 phase blocking and ROS-dependent activation of p21/waf1. Notably, cristacarpin can also be found in *Erythrina burana* Chiov. [37,38,39]. More recently, *E. suberosa* leaf aqueous extract was incorporated into silver nanoparticles and then used in an osteosarcoma cancer cell line (A-431) [40]. The authors discovered that A-431 cell viability was decreased after treatment with this preparation, that also possessed anti-microbial properties, given its strong effects against different pathogenic bacteria and fungi [40].

In addition to cancer studies, *E. suberosa* was tested using different disease models. A work of Serrano, Batista, Bolzani, Santos, Nogueira, Nunes-de-Souza, Latif and Arfan [12] showed that acute treatment per os with the *E. suberosa* alkaloids erysodine and erysothrine produced anxiolytic-like effects in mice tested with two widely used anxiety assays (the elevated plus-maze and the light-dark transition model). Also, the bronchodilator and spasmolytic properties of *E. suberosa* were explored. Indeed, a methanolic crude extract of *E. suberosa* flowers was tested on isolated rabbit jejunum and isolated rabbit tracheal preparations. It was demonstrated that the extract could halt Ca^2+^ channels and showed antioxidant activities, which was revealed by strong scavenging activity on DPPH (2,2-difenil-1-picrylhydrazyl) free radicals. With this last work, the traditional use of *E. suberosa* has been concretely substantiated, however these results should be confirmed using higher models [16].

## 6. Concluding Remarks 

**Figure 3:**
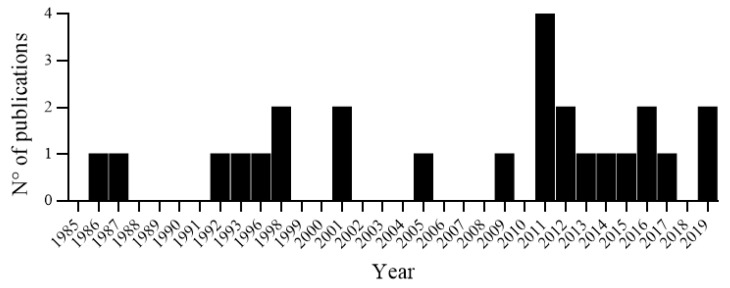
Histogram of publications per year for *Erythrina suberosa* Roxb. from 1985 to 2019 (accessed on 7 October 2019, adapted from Web of Science, https://www.webofknowledge.com).

From its pharmacological properties, phytoconstituents, traditional and common uses of *E. suberosa*, we found that different extracts as well as the isolated compounds can have a robust anticancer biological activity. Still, many efforts should be taken before translating the *E. suberosa* potential into medicinal remedies. There are just a few preclinical studies analysing the effects of *E. suberosa,* more extensive studies are needed (Figure 3). 

This fact should be intended as a concrete stimulus for researchers and clinicians, not a limitation in *E. suberosa* studies. It is expected that new resources, capabilities and energy should be provided for a more in-depth study of *E. suberosa*.
